# Pex11mediates peroxisomal proliferation by promoting deformation of the lipid membrane

**DOI:** 10.1242/bio.201410801

**Published:** 2015-04-24

**Authors:** Yumi Yoshida, Hajime Niwa, Masanori Honsho, Akinori Itoyama, Yukio Fujiki

**Affiliations:** 1 Department of Biology, Faculty of Sciences, Kyushu University Graduate School, 6-10-1 Hakozaki, Higashi-ku, Fukuoka 812-8581, Japan; 2 Graduate School of Systems Life Sciences, Kyushu University Graduate School, 6-10-1 Hakozaki, Higashi-ku, Fukuoka 812-8581, Japan; 3 International Institute for Carbon-Neutral Energy Research (I^2^CNER), Kyushu University, 744 Motooka, Nishi-ku, Fukuoka 819-0395, Japan

**Keywords:** Peroxisomes, Pex11p, Liposomes, Membrane deformation, Amphiphathic helix

## Abstract

Pex11p family proteins are key players in peroxisomal fission, but their molecular mechanisms remains mostly unknown. In the present study, overexpression of Pex11pβ caused substantial vesiculation of peroxisomes in mammalian cells. This vesicle formation was dependent on dynamin-like protein 1 (DLP1) and mitochondrial fission factor (Mff), as knockdown of these proteins diminished peroxisomal fission after Pex11pβ overexpression. The fission-deficient peroxisomes exhibited an elongated morphology, and peroxisomal marker proteins, such as Pex14p or matrix proteins harboring peroxisomal targeting signal 1, were discernible in a segmented staining pattern, like beads on a string. Endogenous Pex11pβ was also distributed a striped pattern, but which was not coincide with Pex14p and PTS1 matrix proteins. Altered morphology of the lipid membrane was observed when recombinant Pex11p proteins were introduced into proteo-liposomes. Constriction of proteo-liposomes was observed under confocal microscopy and electron microscopy, and the reconstituted Pex11pβ protein localized to the membrane constriction site. Introducing point mutations into the N-terminal amphiphathic helix of Pex11pβ strongly reduced peroxisomal fission, and decreased the oligomer formation. These results suggest that Pex11p contributes to the morphogenesis of the peroxisomal membrane, which is required for subsequent fission by DLP1.

## Introduction

Peroxisomes are single membrane-bound organelles that are involved in long-chain fatty acid oxidation, plasmalogen synthesis, and ROS elimination (van den Bosch et al., 1992; Wanders and Waterham, 2006). Peroxisomes are maintained by autonomous proliferation. Peroxisomal proliferation can occur through growth and division of pre-existing peroxisomes or *de novo* synthesis (Lazarow and Fujiki, 1985; Thoms and Erdmann, 2005; Hettema and Motley, 2009). Peroxisomal membrane structure is disrupted in fibroblasts from Zellweger syndrome patients, and this effect can be reversed by introduction of the responsible gene products (Honsho et al., 1998; Matsuzono et al., 1999; South and Gould, 1999; Ghaedi et al., 2000; Muntau et al., 2000; Shimozawa et al., 2000).

Peroxisomes are well known to be maintained by growth and division (Lazarow and Fujiki, 1985). A multi-step reaction for this process has been proposed (Schrader et al., 1998; Koch et al., 2003; Li and Gould, 2003). First, peroxisomal matrix proteins and membrane proteins are imported to peroxisomes. Secondly, peroxisomes increase in size and develop an elongated morphology. Peroxisomal marker proteins were detectable in a segmented pattern on such elongated peroxisomes, like beads on string, with constriction of the membrane (Grabenbauer et al., 2000; Koch et al., 2004; Itoyama et al., 2012; Itoyama et al., 2013). The membrane is then cleaved at the restriction sites to form new peroxisomes. Several proteins involved in this final process have been identified. Dynamin-like protein 1 (DLP1) is a cytosolic large GTPase belonging to the dynamin superfamily (Praefcke and McMahon, 2004). DLP1 is thought to polymerize at the fission site, enabling the membrane to be cleaved by GTP hydrolysis (Yoon et al., 2001; Zhu et al., 2004). Fission 1 (Fis1) and mitochondrial fission factor (Mff) have been identified as the membrane receptor for DLP1 (Kobayashi et al., 2007; Gandre-Babbe and van der Bliek, 2008; Otera et al., 2010; Itoyama et al., 2013). Knockdown or mutation of DLP1, Fis1, and Mff results in fission-deficient, elongated peroxisomes (Koch et al., 2003; Koch et al., 2004; Koch et al., 2005; Tanaka et al., 2006; Kobayashi et al., 2007; Waterham et al., 2007; Gandre-Babbe and van der Bliek, 2008; Otera et al., 2010; Itoyama et al., 2013). These factors were shown to be essential in mitochondrial fission as well as peroxisomal proliferation (Koch et al., 2005; Tanaka et al., 2006; Waterham et al., 2007; Gandre-Babbe and van der Bliek, 2008; Otera et al., 2010). However, spherical peroxisomes require elongation steps prior to fission (Itoyama et al., 2012), which mitochondria do not.

Pex11p family proteins are thought to function in the membrane elongation step (Schrader et al., 1998; Li and Gould, 2002). Pex11p proteins are peroxisomal membrane proteins consisting of three isoforms in mammalian cells, Pex11pα (Abe et al., 1998; Li et al., 2002b), Pex11pβ (Abe and Fujiki, 1998; Schrader et al., 1998; Li et al., 2002a), and Pex11pγ (Li et al., 2002b; Tanaka et al., 2003). Pex11pβ is the most well-characterized isoform, due to its ubiquitous expression and involvement in peroxisomal fission. Overexpression of Pex11pβ results in acceleration of peroxisomal fission, and Pex11pβ knockout mice have a decreased number of peroxisomes (Schrader et al., 1998; Li et al., 2002a; Kobayashi et al., 2007). Pex11pβ is a homo- or hetero-oligomeric protein through an N-terminal amphipathic helix (Kobayashi et al., 2007; Opaliński et al., 2011; Bonekamp et al., 2013). In yeast, a synthetic peptide corresponding to this helical stretch could directly bind to liposomes and disrupt their structure, resulting in tubule-like formations (Opaliński et al., 2011). Pex11pβ is also reported to interact with DLP1 through binding to Fis1 or Mff (Kobayashi et al., 2007; Koch and Brocard, 2012; Itoyama et al., 2013). Introduction of EGFP-tagged or YFP-tagged Pex11pβ promoted peroxisomal fission and resulted in the formation of clusters of elongated peroxisomes (Delille et al., 2010; Koch et al., 2010). Although these data suggest that Pex11pβ has a fundamental role in peroxisomal morphogenesis, the characteristics of Pex11pβ have not been fully elucidated, particularly with respect to its role in membrane deformation.

In the present work, we characterized the role of Pex11pβ in peroxisomal morphogenesis in mammalian cells. The localization of endogenous Pex11pβ was analyzed in fission-deficient cells, which revealed an alternating staining pattern of Pex11pβ and Pex14p/PTS1. The effect of Pex11pβ on the morphology of artificial lipid membranes was also tested. Pex11pβ altered the morphology of reconstituted proteo-liposomes in a manner that depended on the N-terminal amphipathic helix. In this *in vitro* assay, the recombinant Pex11pα and Pex11pγ protein were also effective in morphology of liposomes membrane because of the property of the amphipathic helix. Knockdown of *PEX11β* decreased the number of peroxisomes. In this *in vivo* assay, Pex11pγ had a similar effect on peroxisome formation as Pex11pβ, but Pex11pα had little effects. These results strongly demonstrate that Pex11p functions in the morphogenesis of peroxisomes, particularly at the elongation and constriction steps.

## Results

### Pex11pβ induces peroxisome membrane extension before fission

**Fig. 1.**
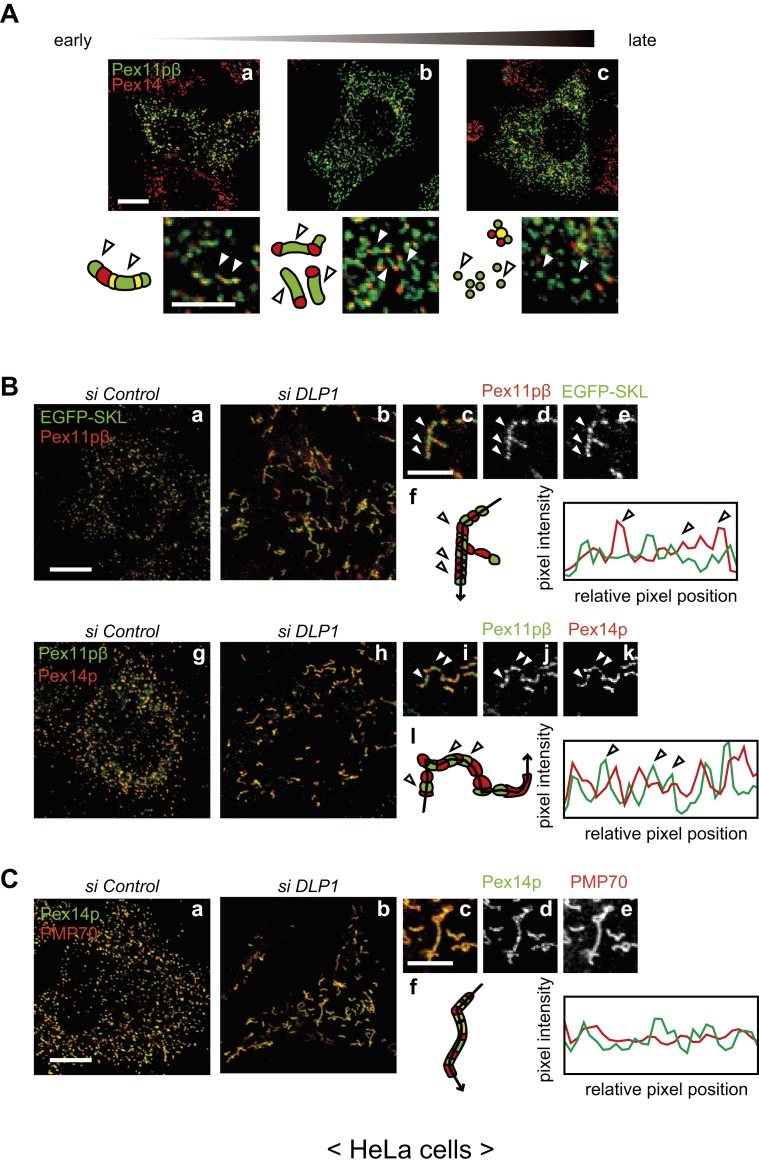
Pex11pβ forms Pex11pβ-enriched membrane subregions on the peroxisomal membrane. (A) HeLa cells were transfected with a plasmid encoding human Pex11pβ for 6–24 h and immunostained with antibodies against Pex11pβ (green) and Pex14p (red). Panels are arranged according to the stage of peroxisomal proliferation. Arrowheads indicate regions enriched in Pex11pβ compared with Pex14p. Scale bars: 10 µm and 5 µm (enlarged). (B) HeLa cells stably expressing EGFP-SKL (a–e) and control HeLa cells (g–k) were treated with a control siRNA (a,g) or *DLP1* siRNA (b–e,h–k) for 72 h. Cells were subjected to immunostaining using the anti-Pex11pβ antibody (a–e) or double-stained with the anti-Pex11pβ antibody and an anti-Pex14p antibody (g–k). Magnified view of *DLP1* siRNA-treated cells (c–e,i–k), and pixel intensity by line scanning along the elongated peroxisome (arrow) were plotted (f,l). Clear peaks of Pex11pβ were highlighted by arrowheads. Scale bars: 10 µm and 5 µm (enlarged). (C) HeLa cells were treated with a control siRNA (a) or *DLP1* siRNA (b) for 72 h, and stained the *DLP1* siRNA-treated cells with antibodies to Pex14p and PMP70 in magnified views (c–e). Line scanning of elongated peroxisomes was shown in (f). Scale bars: 10 µm and 5 µm (magnified view).

Pex11pβ induces peroxisomal fission when overexpressed in mammalian cells. To assess the effect of Pex11pβ on peroxisomal morphology, we overexpressed untagged Pex11pβ in HeLa cells (Fig. 1A). For the detection of untagged Pex11pβ protein, an anti-Pex11pβ antibody was raised against the N-terminus, which encompasses amino acid residues 1–153 of human Pex11pβ (Itoyama et al., 2012). The anti-Pex11pβ antibody specifically recognized endogenous Pex11pβ in HeLa cells both by western blotting and by immunostaining (supplementary material Fig. S1). At an early stage of peroxisomal fission, peroxisomes were extended, and Pex11pβ-enriched membrane subregions were generated (Fig. 1Aa,b, arrowheads). These subregions were then pinched off to form small vesicles in which Pex11pβ was concentrated (Fig. 1Ac). This vesicle formation depended on peroxisomal fission factors, such as DLP1 and Mff, because the fission did not occur in cells treated with siRNA against such fission factors (supplementary material Fig. S2A). Overexpression of Pex11pβ caused extremely extension of peroxisomes in the fission-deficient cells (supplementary material Fig. S2Eb).

### Endogenous Pex11pβ forms membrane subregions on peroxisomes

To examine whether endogenous Pex11pβ forms specialized membrane subregions on peroxisomes, *DLP1* was knocked down in HeLa cells stably expressing EGFP tagged with peroxisomal targeting signal 1 sequence (Ser-Lys-Leu) at the C-terminus (EGFP-SKL), and the cells were immunostained with the anti-Pex11pβ antibody (Fig. 1Ba–e). Peroxisomes showed the typical extended morphology, as detected by EGFP-SKL fluorescence. Endogenous Pex11pβ was detected in regions in which PTS1 intensity was low (arrowheads). Line scanning of elongated peroxisomes clearly showed that the peak of the Pex11pβ signal did not overlap with that of the EGFP-SKL signal (Fig. 1Bf). The localization of Pex11pβ was compared with Pex14p in the same way (Fig. 1Bg–l). Both Pex11pβ and Pex14p showed a granular staining pattern along the peroxisomes. Pex14p and Pex11pβ localized mutually exclusive regions on elongated peroxisome structures (Fig. 1Bi–k), the distribution of Pex11pβ alternated with that of Pex14p by line scanning (Fig. 1Bl). By contrast, another peroxisomal membrane protein, PMP70, showed uniform distribution on elongated peroxisomes (Fig. 1C). These results indicate that Pex11pβ-enriched subregions on the peroxisomal membrane form before the fission process is initiated.

### Reconstitution of Pex11pβ into proteo-liposomes

**Fig. 2.**
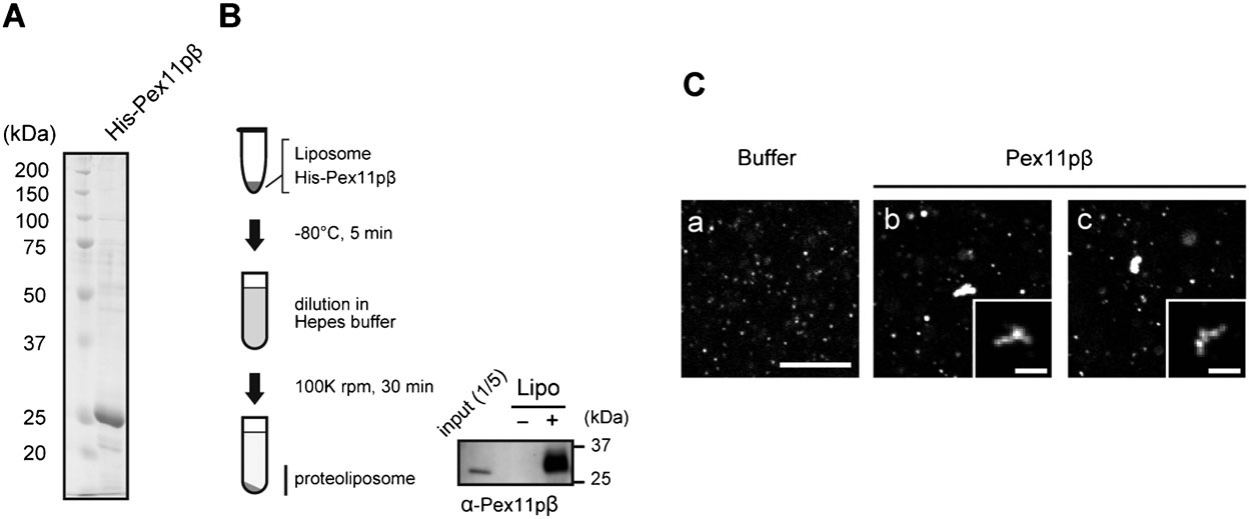
Reconstitution of recombinant Pex11pβ into proteo-liposomes. (A) Coomassie Brilliant Blue-stained SDS-PAGE gel of recombinant His-Pex11pβ protein. (B) Schematic model of incorporation into proteo-liposomes. Purified Pex11pβ was snap-frozen in the presence or absence of liposomes and the mixture was then thawed and diluted in Hepes-KOH buffer. Proteo-liposomes were collected by centrifugation, and incorporation of Pex11pβ was evaluated by western blotting using the anti-Pex11pβ antibody. (C) Pex11pβ was reconstituted into rhodamine-labeled proteo-liposomes, which were observed under confocal microscopy (b,c). Liposomes were also snap-frozen with the buffer used in the purification of Pex11pβ, as a control (a). Confocal micrographs were also taken under short-time exposure and enlarged (inset). Scale bars: 10 µm and 5 µm (inset).

**Fig. 3.**
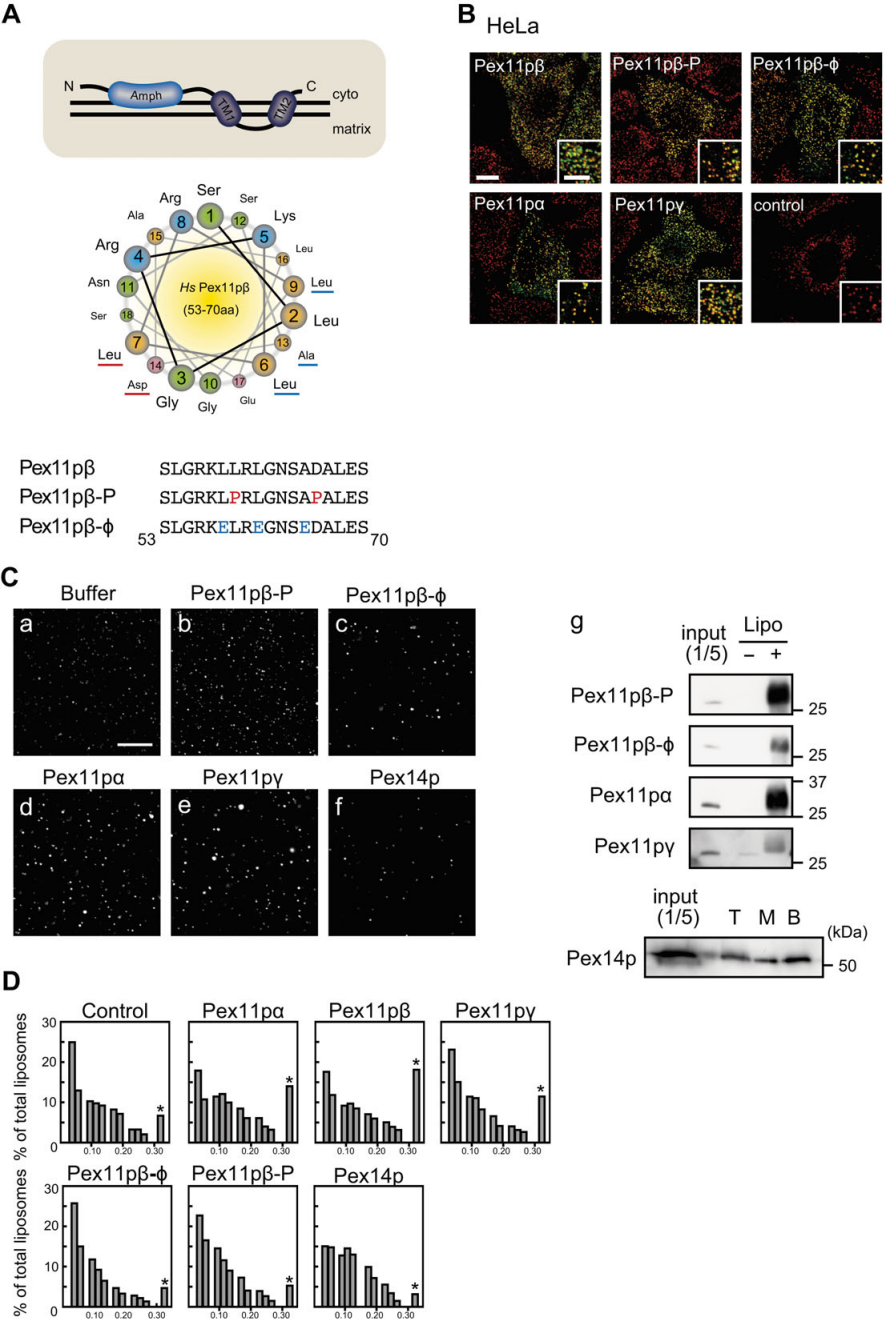
The effect of the N-terminal amphipathic helix of Pex11pβ on the morphology of reconstituted liposomes. (A) Molecular structure and helical wheel representation of the region comprising the amphipathic helix encompassing the amino acid residues at positions 53–70 of Hs-Pex11pβ. The helical wheels are presented by looking down into the helical axis and taking Ser55 as the first amino acid residue with the largest circle, the second residue with the second “largest”, followed by those with smaller and smaller sizes. Amino acids are colored according to the physicochemical properties of the side chains (hydrophobic – yellow; polar, positively charged – blue; polar, negatively charged – pink; polar, uncharged – green). The hydrophobic surface was mutated to glutamate (indicated in blue) and proline (indicated in red). (B) Myc-tagged Pex11p proteins were overexpressed in HeLa cells and immunostained with antibodies to Myc (green) and Pex14p (red), respectively (a–f). Peroxisomal fission was observed under confocal microscopy. Scale bars: 10 µm and 5 µm (inset). (C) Confocal micrographs of control liposomes (a) and proteo-liposomes which were reconstituted with Pex11pβ-P (b), Pex11pb-φ (c), Pex11pα (d), Pex11pγ (e), or Pex14p (f). Scale bar: 10 µm. Incorporation of recombinant protein into liposomes was evaluated by western blotting (g). (D) Sizes of liposomes were quantitated, and the size distribution is represented as histograms. The rightmost bar (*) indicates total percentage of over 0.3 µm^2^. At least 1000 particles from randomly selected fields were counted.

Pex11pβ has an amphiphathic helix in its N-terminal region (Fig. 3A, upper panel). A synthetic peptide consisting of this region caused deformation of negatively charged liposomes into tubular extensions in yeast (Opaliński et al., 2011). To analyze the activity of the amphiphathic helix in mammals, recombinant human full-length Pex11pβ protein was purified. Deletion of the C-terminal nine amino acids of the cytosolic region gave rise to better expression of Pex11pβ in *E. coli* (Fig. 2A), and had the same potency of Pex11pβ in inducing peroxisome fission *in vivo* (supplementary material Fig. S2B,C) (Kobayashi et al., 2007). Therefore, the Pex11pβΔC protein was used as an intact Pex11pβ in *in vitro* assays. The purified Pex11pβ protein was reconstituted into proteo-liposomes as described in Fig. 2B. Because Pex11pβ protein was solubilized in sodium N-lauroylsarcosine, this detergent solution was used in control experiments. Affinity-purified Pex11pβ protein was mixed with rhodamine-labeled liposomes and then frozen at −80°C for 5 min. Then, the mixture was diluted in Hepes-KOH buffer and ultracentrifuged to concentrate the liposomes. This freeze-thaw process was also performed in the absence of liposomes as a control. Pex11pβ was efficiently incorporated into liposomes, as detected by western blotting using the anti-Pex11pβ antibody (Fig. 2B; supplementary material Fig. S3). To determine the morphological effect, reconstituted liposomes were observed under confocal microscopy (LSM-710; Zeiss). In the N-lauroylsarcosine-treated control condition, liposomes were small and uniformly dispersed (Fig. 2Ca), while Pex11pβ-reconstituted proteo-liposomes formed large clusters (Fig. 2Cb,c). Pex11pβ-liposomes appeared to consist of aggregates of small liposomes in images taken with a short exposure (Fig. 2C, inset).

### The N-terminal cytosolic region of Pex11pβ is responsible for its activity

Our group earlier showed that the Pex11pβ N-terminal cytosolic region is required for its ability to enhance peroxisomal fission (Kobayashi et al., 2007), and another group has reported that a synthetic peptide corresponding to the N-terminal amphipathic helix of Pex11pβ induces the formation of tubule-like structures from artificial membranes *in vitro* (Opaliński et al., 2011). This membrane-deforming activity was shown to be dependent on the amphipathic properties of the Pex11pβ α-helical structure by introducing point mutations into its sequence. We therefore made the corresponding mutant forms of human Pex11pβ. The region to be mutated was chosen by measurement of the hydrophobic moment using Heliquest (see Materials and Methods). Negatively charged amino acids were introduced into the hydrophobic surface of the amphipathic region to change the overall charge of this region without affecting its α-helical structure (Pex11pβ-φ) or proline residues were also introduced to hamper the formation of the α-helix (Pex11pβ-P) (Fig. 3A). Using multiple sequence alignment of Pex11p proteins from different species and the three human isoforms, the amphipathic helices in human Pex11pα, Pex11pβ, and Pex11pγ were determined (supplementary material Fig. S4A, underline). The average of the hydrophobic moment of this region was 0.342 in Pex11pα, 0.359 in Pex11pβ, and 0.501 in Pex11pγ (supplementary material Fig. S4B, black line). To confirm the effect, the point-mutated Myc-Pex11pβ-φ and Myc-Pex11pβ-P were overexpressed in HeLa cells, and the cells were subjected to immunostaining using antibodies to Myc and Pex14p (Fig. 3B). While the wild-type Myc-Pex11pβ enhanced peroxisomal fission as shown in Fig. 1A, overexpression of the mutated forms did not change the number of peroxisomes in HeLa cells (supplementary material Fig. S2C). The effect of Pex11pα and Pex11pγ on peroxisomal fission was also examined. Both Pex11p isomers had weak effect on the fission. The fission properties of the series of Pex11p proteins were also tested in cells in which *DLP1* had been knocked down. The wild-type Pex11pβ and Pex11pγ were incorporated into peroxisomes, forming Myc-Pex11p-enriched regions and enhancing peroxisomal elongation overall, while the Pex11pβ mutants and Pex11pα did not result in additional elongation of peroxisomes (supplementary material Fig. S2E). Then, as the wild-type Pex11pβ (Fig. 2C), the mutants, Pex11pα, and Pex11pγ proteins were purified and reconstituted into proteo-liposomes to determine the effect of these proteins on the liposomal morphology. As a control experiment, a typical peroxisomal membrane protein, Pex14p, was also examined. Pex14p was heavily precipitated in the absence of liposomes so that the reconstitution was carried out by the floating method as shown in supplementary material Fig. S3. Under confocal microscopy, all of the Pex11p isomers showed liposomal aggregation activity to some extent, while the Pex11pβ mutants and Pex14p had no effect on the reconstituted proteo-liposomes (Fig. 3Ca–f). Reconstitution of these proteins was evaluated by western blotting in precipitated liposomes (Fig. 3Cg). The size distribution histogram of all reconstituted proteo-liposomes is shown in Fig. 3D. The percentage of aggregated liposomes (greater than 0.3 µm^2^) dramatically increased when Pex11pβ was reconstituted into proteo-liposomes. An increase was similarly observed in proteo-liposomes reconstituted with Pex11pα and Pex11pγ. Pex14p and the mutant forms of Pex11pβ had no effect on the percentage of large liposomal clusters.

### Pex11pβ forms clusters in reconstituted liposomes

**Fig. 4.**
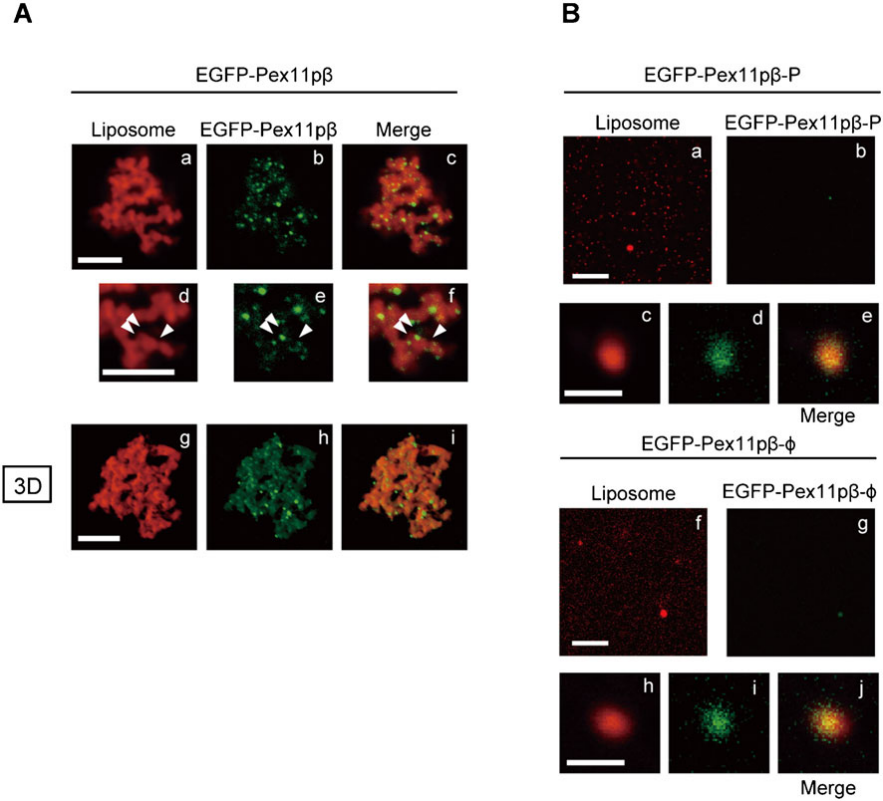
Localization of Pex11p protein in reconstituted proteo-liposomes. (A) EGFP-tagged Pex11pβ protein was introduced into rhodamine-labeled liposomes, which were observed under confocal microscopy (a–f). Photos were enlarged in panels d–f. Arrowheads indicate clusters of Pex11pβ on the proteo-liposomes. Image stacks were collected along the z-axis and rendered as maximum intensity projections using Zen 2011 software (Carlzeiss) (g–i). Scale bars: 5 µm and 2 µm (enlarged). (B) Rhodamine-labeled liposomes were reconstituted with EGFP-fused mutants, Pex11pβ-P (a–e) and Pex11pβ-φ (f–j). Close-up views of the proteo-liposomes for Pex11pβ-P (c–e) and Pex11pβ-φ (h–j). Scale bars: 10 µm and 1 µm (close-up view).

Endogenous Pex11pβ was accumulated to specific membrane subregions in fission-deficient peroxisomes (see Fig. 1B). Next, we tested whether Pex11pβ forms these structures in proteo-liposomes reconstituted with Pex11pβ. To assess this, rhodamine-labeled liposomes were reconstituted with MBP-EGFP-Pex11pβ (hereafter termed EGFP-Pex11pβ, see Materials and Methods) and subjected to confocal microscopy. As the same as that of untagged Pex11pβ, EGFP-Pex11pβ reconstituted liposomes connected each other and formed large aggregation. On the liposome aggregates, dots of EGFP signal were observed at the edge of each spherical structure (Fig. 4Aa–f). EGFP signal should be observed at the contact sites between liposomes, as if EGFP-Pex11pβ connected the vesicles. Z-stack images were also taken and the maximal intensity projections are shown (Fig. 4Ag–i). Pex11pβ localized to striated structures in addition to the contact sites between small liposomes. On the other hand, both EGFP-tagged mutant forms of Pex11pβ failed to cluster on the reconstituted proteo-liposomes (Fig. 4B).

### Clusters of Pex11pβ-proteo-liposomes show a continuous membrane

**Fig. 5.**
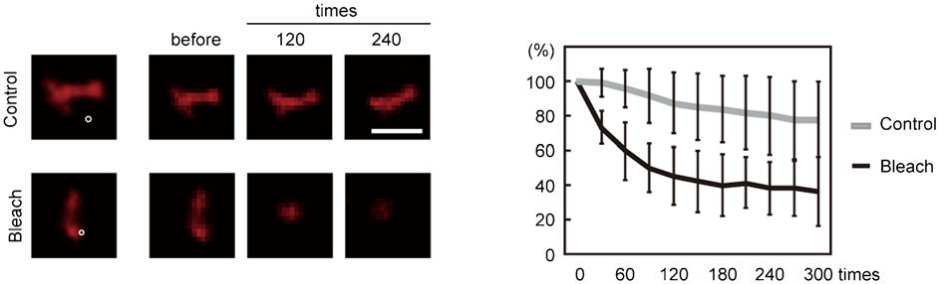
Membrane continuity of Pex11pβ-containing proteo-liposomes. A small region (white circle) of Pex11pβ-reconstituted proteo-liposomes was repeatedly photobleached. Images were taken before and after 30 rounds of photobleaching. In the control experiment, an adjacent region of proteo-liposomes was photobleached. The fluorescence intensity of the liposomes was measured and normalized to the intensity prior to photobleaching. Data represent the means±SD. Scale bar: 2 µm.

Next, a photobleaching assay was used to assess whether the clustered liposomes formed by reconstituting proteo-liposomes with Pex11pβ are aggregates of independent liposomes or continuous liposomes. A small fraction of clustered liposomes was repeatedly photobleached, and images were taken every 30 rounds of photobleaching (Fig. 5). In the control experiment, an adjacent region was repeatedly photobleached, and the area was scanned after 300 rounds of photobleaching. After the control experiment, the original liposomal cluster was re-examined. As shown in Fig. 5, the signal disappeared after repeated photobleaching of a small region. The total fluorescent intensity of the liposomes was measured and graphed after it was normalized to the initial florescence intensity. The fluorescence signal of the entire area decreased after photobleaching of a small region of liposomes. These results suggest that the liposomes consist of a continuous membrane rather than a cluster of small liposomes.

### Electron microscopic analysis of Pex11pβ-reconstituted proteo-liposomes

**Fig. 6.**
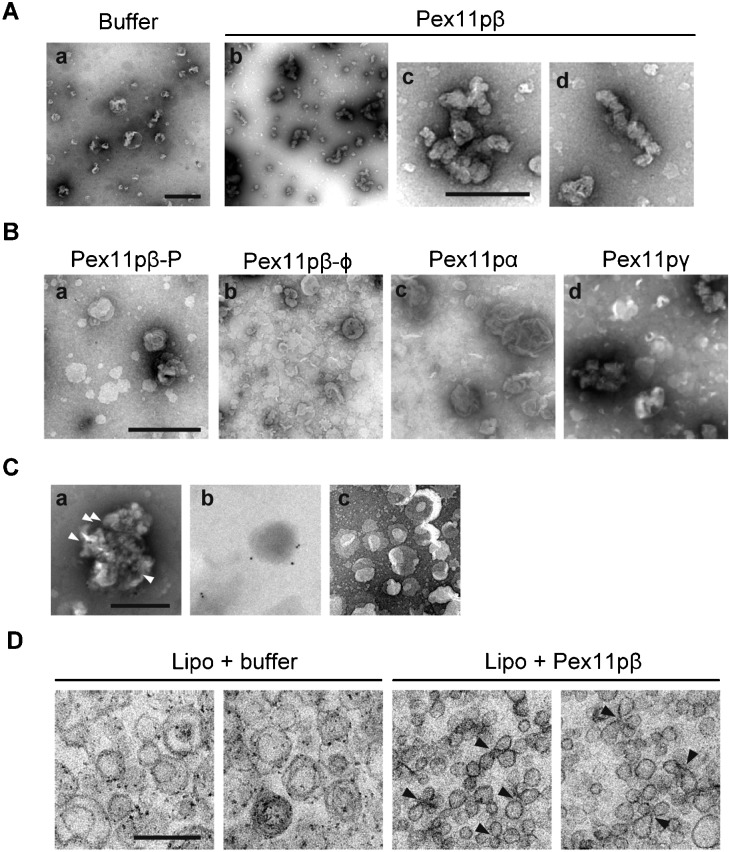
Morphology of Pex11pβ-reconstituted proteo-liposomes. (A–C) Negative staining electron micrographs of Pex11pβ-reconstituted proteo-liposomes. (A) Control liposomes (a) and Pex11pβ-reconstituted proteo-liposomes (b–d) were negatively stained with 2% uranyl acetate. Pex11pβ-reconstituted liposomes are magnified in panels c and d. Scale bars: 1 µm (a,b) and 0.5 µm (c,d). (B) Negative staining of proteo-liposomes reconstituted with Pex11pβ-P (a), Pex11pβ-Φ (b), Pex11pα (c), or Pex11pγ (d). Scale bar: 1 µm. (C) Reconstituted proteo-liposomes were subjected to immune labeling, and stained with uranyl acetate. Pex11pβ- (a) or Pex11pβ-P- (b) reconstituted proteo-liposomes were sequentially incubated with the anti-Pex11pβ antibody and an anti-rabbit IgG conjugated to 10 nm gold particles. The anti-Pex11pβ antibody and gold-labeled anti-rabbit IgG did not react with the control liposomes (c). Scale bar: 0.5 µm. Arrowheads indicate the gold particles (see text). (D) The reconstituted proteo-liposomes were subjected to electron microscopy. Control-treated liposomes (left) and Pex11pβ-reconstituted liposomes (right) were pelleted by ultracentrifugation and prepared for embedding and ultra-thin sectioning as described in the Materials and Methods. Note that the constriction of the liposomal membrane was observed in Pex11pβ-reconstituted liposomes (arrowheads). Scale bar: 0.2 µm.

To observe the morphology of the liposomes in detail, negative staining electron microscopy was used. Control treatment of liposomes had no effect on their morphology, while reconstitution of liposomes with Pex11pβ caused clustering of small vesicles, as observed under confocal microscopy (Fig. 6Aa–d). When Pex11pγ was incorporated into liposomes, liposomes formed small aggregates (Fig. 6Bd). Mutant forms of Pex11pβ had no effect on the clustering of liposomes (Fig. 6Ba,b), however, Pex11pα reconstituted liposomes slightly expanded (Fig. 6Bc), consistent with the results shown in Fig. 3C,D. The localization of reconstituted Pex11pβ was detected by immunostaining using the anti-Pex11pβ antibody and immunogold labeling. Gold particles were detected at the contact sites between small vesicles in Pex11pβ-reconstituted liposomes (Fig. 6Ca, arrowheads), while immunogold was detected on the smooth surface of small liposomes in Pex11pβ-P-reconstituted liposomes (Fig. 6Cb). Control liposomes showed no significant signal (Fig. 6Cc). Next, reconstituted liposomes were subjected to ultra-thin electron microscopy (Fig. 6D). Control liposomes and Pex11pβ-reconstituted liposomes were concentrated by ultracentrifugation, then fixed with glutaraldehyde and embedded in Epon. In control samples, small liposomes were dispersed separately. On the contrary, in the Pex11pβ-reconstituted samples, small clusters of connected liposomes were observed. Liposomes were associated with each other, and appeared to be constricted at the contact sites.

### Pex11p affects the number of peroxisomes in HeLa cells without inhibiting matrix protein import

**Fig. 7.**
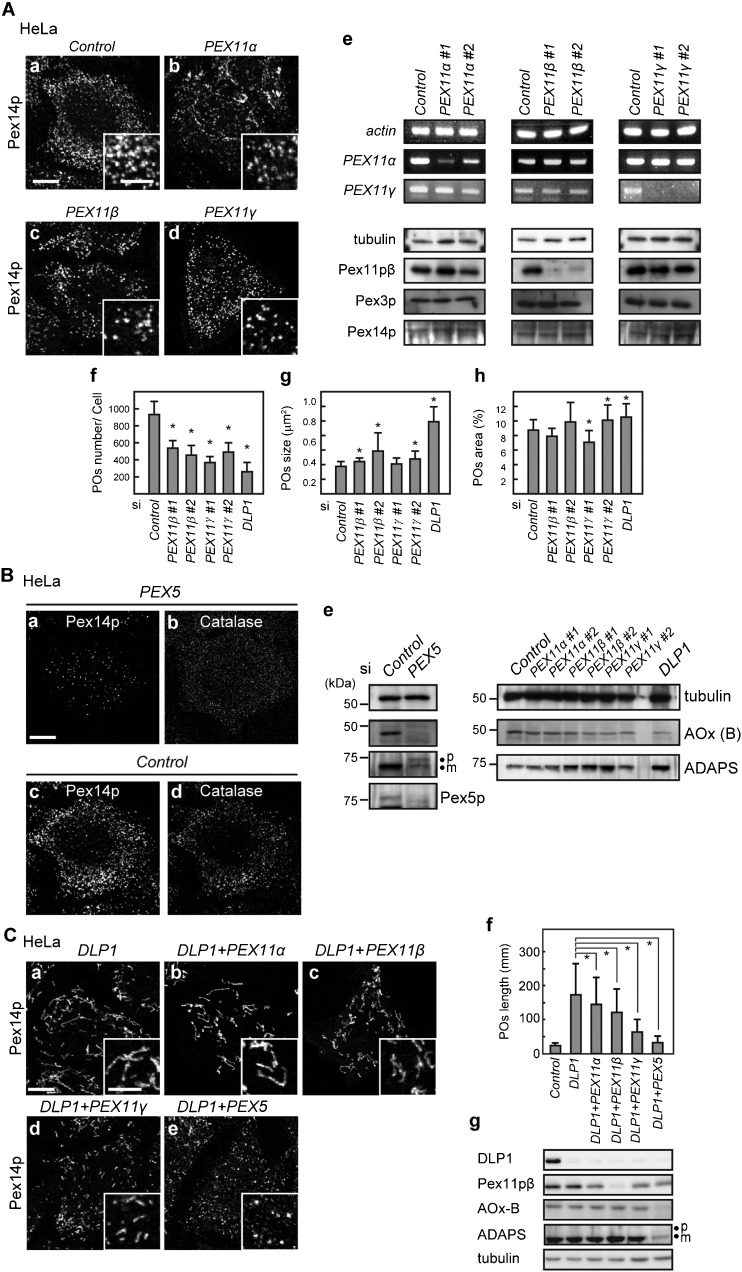
Knockdown of *PEX11* in HeLa cells. (A) HeLa cells were treated for 96 h with a control siRNA (a) or siRNA for *PEX11α* (b), *PEX11β* (c), or *PEX11γ* (d). Cells were then immunostained with an anti-Pex14p antibody. Scale bars: 10 µm and 5 µm (inset). The efficiency of knockdown was evaluated by RT-PCR (e, upper panel) and western blotting (e, lower panel). *PEX11α* and *PEX11γ* mRNA levels were assessed by RT-PCR using total RNA. Actin was used as a loading control. Pex11pβ protein levels were verified by immunoblot analysis using the antibody to Pex11pβ. Peroxisomal marker proteins, Pex3p and Pex14p, were also assessed. Tubulin was used as a loading control. Peroxisomal abundance per cell (f), average peroxisomal size (g), and peroxisomal area per cell (h) in each siRNA-treated cell type were determined as described in the Materials and Methods. At least 20 cells were randomly selected and analyzed at each time point. Data represent the means±SD. *p<0.05. (B) HeLa cells were treated with a control siRNA or siRNA for *PEX5* for 48 h and immunostained with antibodies to Pex14p (a,c) and catalase (b,d). Scale bar: 10 µm. Cells were lysed and analyzed by SDS-PAGE and immunoblotting using the antibodies indicated on the right in the panel (e). ‘p’ and ‘m’ in the panel of ADAPS indicates the larger ADAPS precursor harboring PTS2 and its mature form, respectively. (C) HeLa cells were transfected for 96 h with siRNAs each for *DLP1* (a) in combination with the indicated genes (b–e). Cells were then subjected to immunostaining with an anti-Pex14p antibody. Scale bars: 10 µm and 5 µm (inset). The average lengths of peroxisomes of the cells were measured and plotted in (f). Cells were lysed and analyzed by SDS-PAGE and immunoblotting using the antibodies indicated on the left in the panel (g). Data represent the means±SD. *p<0.05.

To analyze the Pex11p function *in vivo*, the morphology and number of peroxisomes in HeLa cells that had been transfected with siRNAs targeting *PEX11α*, *PEX11β*, or *PEX11γ* were compared to those of control and *DLP1* siRNA-treated cells. Treatment of HeLa cells with siRNA targeting *PEX11α* or *PEX11γ* for 96 h resulted in significant decreases in *PEX11α* and *PEX11γ* mRNA, respectively (Fig. 7Ae). Similarly, the levels of Pex11pβ protein were reduced to below the level of detection by western blotting in HeLa cells transfected with siRNA for *PEX11β*. The morphology of peroxisomes was mostly unaffected in cells in which *PEX11β* or *PEX11γ* was knocked down, in contrast to the cells in which *DLP1* was knocked down (Fig. 7Aa–d,Ca). However, the number of peroxisomes per cell was decreased, especially in cells treated with *PEX11β* or *PEX11γ* siRNA (Fig. 7Af). On the other hand, the average peroxisome size tended to increase in response to knockdown of *PEX11β* or *PEX11γ* (Fig. 7Ag). Evaluation of peroxisomal area revealed no obvious trend (Fig. 7Ah). In *PEX11α* siRNA-treated cells, peroxisomal aggregation was often observed, which made it difficult to determine the precise size and number of peroxisomes; these results were therefore excluded from the graph.

Defects in PTS1-mediated peroxisomal matrix protein import affect the number of peroxisomes in human fibroblasts (Chang et al., 1999), and CHO cells (Otera et al., 2002). We therefore investigated whether *PEX11* knockdown impedes peroxisomal matrix protein import. Matrix PTS1-proteins are imported into peroxisomes through binding with their cytosolic receptor, Pex5p. In *PEX5* siRNA-treated HeLa cells, peroxisomal localization of catalase, a PTS1-type protein, was disrupted (Fig. 7Ba,b), and the maturation pattern of matrix proteins by western blotting showed the typical pattern seen in import-deficient cells (Fig. 7Be, left). In such cells, the number of peroxisomes was greatly decreased, without elongation as assessed with Pex14p-positive peroxisomes (Fig. 7Ba). In comparison with knockdown of *PEX5*, knockdown of *PEX11* resulted in normal peroxisomal matrix protein import and processing as in control siRNA-treated cells (Fig. 7Be, right). These results confirm that Pex11pβ functions in the morphogenesis of peroxisomes, as has been previously established.

The effect of Pex11p on morphogenesis was tested in *DLP1* siRNA-treated cells, because reconstitution with Pex11p resulted in elongated peroxisomes. When cells were treated with siRNA for *PEX11* in combination with *DLP1* siRNA, shorter peroxisomes were observed (Fig. 7Ca–d,f). Similar to the single-knockdown cells, any of the *PEX11*- and *DLP1*-double knockdown cells show normal in peroxisomal matrix protein import (Fig. 7Cg). Together these results suggested that peroxisomal elongation was inhibited in the *PEX11*- and *DLP1*-double knocked down cells.

## Discussion

The Pex11p family of proteins has been identified as peroxisome-specific fission factors (Abe et al., 1998; Schrader et al., 1998; Li et al., 2002a; Li and Gould, 2002; Tanaka et al., 2003). Pex11pβ increases the number of peroxisomes when overexpressed in cultured cells, while Pex11pβ knockout mice have fewer peroxisomes (Li et al., 2002a). Pex11pβ has also been implicated in docosahexaenoic acid (DHA)-induced peroxisomal fission in acyl-CoA oxidase (AOx)-deficient cells (Itoyama et al., 2012). However, the function of Pex11pβ at a molecular level is poorly understood.

We therefore set out to characterize the localization of Pex11pβ in mammalian cells. Both endogenous and exogenous Pex11pβ were enriched within subregions of the peroxisome membrane (Fig. 1A,B). Pex11pβ was segregated from other peroxisomal marker proteins in the absence of DLP1. Elongated peroxisomes showed alternating staining for Pex11pβ and Pex14p/PTS1 (Fig. 1B), which is consistent with previous findings in a Pex11pβ overexpression system (Schrader et al., 1998; Li et al., 2002a; Kobayashi et al., 2007). Exogenously expressed Pex11pβ-Myc has been reported to induce peroxisomal tubules with PMP70-positive globular termini (Schrader et al., 1998). Similarly, EGFP-tagged Pex11p family proteins and C-terminally YFP-tagged Pex11pβ proteins form tubular peroxisomal membrane compartments due to the inhibition of peroxisomal division (Kobayashi et al., 2007; Delille et al., 2010; Koch et al., 2010). Here, we demonstrate that overexpressed untagged or Myc-tagged Pex11pβ was incorporated into Pex11pβ-enriched membrane tubules, and small vesicles were generated by the fission of these tubules (Fig. 1A; supplementary material Fig. S2B–E). This reflects the tendency of peroxisome fission to occur at the Pex11pβ-enriched regions, suggesting that these points are cleavage sites for DLP1 during the process of fission.

The mechanism through which Pex11pβ mediates peroxisomal cleavage is unknown. It is possible that this process occurs through membrane deformation by Pex11pβ. DLP1 belongs to the dynamin family of proteins, which possess self-oligomerization activity (Praefcke and McMahon, 2004). DLP1 and other members of this protein family can form sedimentable higher-order oligomers (Yoon et al., 2001; Tanaka et al., 2006; Chang et al., 2010) and possess liposome-tubulation activity (Yoon et al., 2001). This oligomerization and tubulation property is essential for membrane scission by DLP1 *in vivo* and *in vitro* (Yoon et al., 2001; Waterham et al., 2007; Chang et al., 2010). In clathrin-mediated endocytosis, cytosolic adapter proteins harboring lipid-binding domains [i.e., epsin N-terminal homology (ENTH) domains, Bin–Amphiphysin–Rvs (BAR) domains, and N-terminal BAR (N-BAR) domains] bend the plasma membrane prior to membrane fission by dynamin-1 (Takei et al., 1999; Ford et al., 2002; Itoh et al., 2005). These adaptor proteins can polymerize at the lipid membrane, and induce narrow lipid tubules *in vitro* and *in vivo* (Shupliakov et al., 1997; Wu et al., 2010). Dynamin was preferentially recruited onto high-curvature lipid membranes, suggesting that accumulated dynamin-1 provides efficient adaptor protein support at the fission site (Takei et al., 2001; Yoshida et al., 2004).

In our study, proteo-liposomes reconstituted with recombinant His-Pex11pβ exhibited changes in morphology (Figs 2–4,6,7). Membrane constriction was observed after reconstitution of Pex11pβ recombinant protein into liposomes. Similar to our results, constriction of peroxisomes was reported in response to *DLP1* knockdown (Koch et al., 2004). The ultrastructure of peroxisomes in HepG2 cells treated with *DLP1* siRNA revealed a constricted but interconnected arrangement, like beads on a string. The pattern of the GFP-PTS1 signal in these cells was segmented, consistent with our observations (Fig. 1B). An alternating pattern of endogenous Pex11pβ and PTS1 suggests that Pex11pβ may be concentrated at the constricted region in the elongated peroxisomes. EGFP-Pex11pβ and Pex11pβ were localized to the connection sites of reconstituted liposomes, resembling the localization of endogenous Pex11pβ (Figs 1,4,6). In accordance with the expected role of Pex11pβ, these data provide further evidence to suggest that Pex11pβ form peroxisomal fission sites prior to the actions of downstream fission factors.

Recently, another group has demonstrated that a synthetic peptide comprising the N-terminal amphipathic helix of yeast Pex11p caused liposomes to form tubule-like structures (Opaliński et al., 2011). This activity was required for peroxisomal division in *H. polymorpha*. In our studies testing human full-length Pex11pβ protein, the function was evidently established in mammalian cells too, as verified by introduction of a point mutation into this region changed the activity of Pex11pβ *in vivo* and *in vitro* (Figs 3,4). However, we are also interpreted these results to mean that the impaired activity was due to not only a defect in the amphiphathic properties of the helix for the membrane but also a disruption in its ability to form oligomers, because the mutant forms of Pex11pβ lacking the fission activity showed a reduced level of oligomer formation, as compared to that of the with-type (supplementary material Fig. S2F, open arrowheads). In our current and earlier studies, oligomer formation of Pex11pβ was required for its activity (supplementary material Fig. S2B–E) (Kobayashi et al., 2007; Itoyama et al., 2012). N-terminal cytosolic extension of Pex11pβ was indispensable for oligomer formation and peroxisomal fission. Moreover, MBP-Pex11pβ could oligomerize within DHA-enriched liposomes, which resembled normal peroxisomes in lipid composition, but not in oleic acid-enriched liposomes, which resembled proliferation-deficient peroxisomes (Itoyama et al., 2012). Consistently, proto-liposomes reconstituted with His-Pex11pβ exhibited an altered morphology when DHA-PC and DHA-PE were used as a starting material (Figs 2–7). BAR and N-BAR domains contain an amphiphathic helix (Dawson et al., 2006). The crystal structure revealed that their crescent-like structure and the surface distribution of positively charged amino acids were required for membrane tubulation (Peter et al., 2004). Anti-parallel homo-dimers form a concave surface with a diameter of 22 nm. This structure can stabilize membrane tubules of particular sizes, which may regulate the endocytic machinery in a spatio-temporal manner. As the similar scenario may be true for mammalian Pex11p, the active oligomer was detected by Blue Native PAGE in all isomers of Pex11p (supplementary material Fig. S2F, open arrowheads), corresponding to the oligomer shown in a glycerol gradient with purified protein (Itoyama et al., 2012). How this oligomer is oriented within the lipid membrane *in vivo* and *in vitro* is unclear, but the Pex11pβ-P and Pex11pβ-φ mutants form higher-order oligomers or aggregates in Blue Native PAGE (supplementary material Fig. S2F, solid arrowheads), suggesting that the proper oligomerization of Pex11pβ in a manner dependent on its N-terminal amphiphathic region is essential for tubulation on lipid membranes.

The Pex11p family of proteins contains three isoforms, namely Pex11pα, Pex11pβ, and Pex11pγ. In our *in vivo* experiments, the activity of Pex11pβ and Pex11pγ was similar, and Pex11pα showed less activity. The former two isoforms induced peroxisomal fission when overexpressed (Fig. 3B; supplementary material Fig. S2C–F). While Pex11pα showed little activity, peroxisomal abundance was decreased in cells in which *PEX11α* was knocked down because of the formation of large peroxisome aggregates (Fig. 7A). This aggregation was similar in structure to juxtaposed elongated peroxisomes (JEP). JEPs form from an imbalance in Pex11p family proteins and other fission factors (Delille et al., 2010; Koch and Brocard, 2012). Pex11pα may balance with the activity of the other active isoforms, Pex11pβ and Pex11pγ.

Recently, it was suggested that Pex11pγ may be involved in matrix protein import in Pex11pβ-deficient human fibroblasts (Ebberink et al., 2012). The expression level of Pex11pγ in patient cells decreased when cells were cultured at 40°C. In our assays, no obvious defect in matrix protein import was observed in cells in which *PEX11γ* was knocked down. Additional *PEX5* knockdown countered the effect of peroxisomal elongation induced by *DLP1* siRNA (Fig. 7Cf), and major peroxisomal matrix proteins were not imported (Fig. 7Cg). On the other hand, the shorter peroxisomes were actually observed but less in cells in which both *DLP1* and *PEX11γ* were knocked down (Fig. 7Cd), as compared to cells in which only *DLP1* was knocked down (Fig. 7Cf). Peroxisomal PTS1- and PTS2-proteins were normally imported in the double knocked down cells (Fig. 7Cg). These results suggest that Pex11pγ plays a role in peroxisomes morphogenesis, not matrix protein import.

**Fig. 8.**
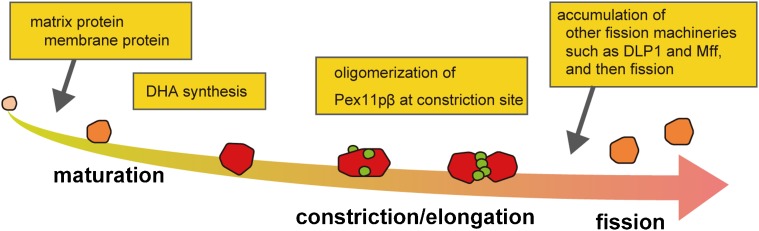
A schematic model for the Pex11pβ function in peroxisomal fission. Schematic drawing of peroxisomes during fission process as maturation was represented in red. The whole process consists of three steps, maturation, constriction/elongation, and fission. Pex11pβ is highlighted in green only in the constriction/elongation step, where it locates at the constriction sites. Note that the Pex11pβ forms the active oligomer at the stage to deform peroxisomal membrane.

In conclusion, Pex11pβ protein functions in peroxisomal morphogenesis by deforming the peroxisomal membrane. Based on these data and our previous observations, we proposed a model of the Pex11pβ function in peroxisomal fission, as illustrated in Fig. 8. First, immature peroxisomes are derived by growth and division from mature peroxisomes and/or *de novo* formation. Such peroxisomes then get matured after the import of matrix and membrane proteins. Peroxisomal fission is initiated with DHA (Itoyama et al., 2012). Incorporation of DHA into presumably phospholipids in peroxisomal membrane gives rise to the Pex11pβ oligomerization to form subregions enriched in Pex11pβ on the membrane, and constriction sites on the mature peroxisomes. Pex11pβ forms the active oligomer and tightly interacts with the membrane through the amphiphathic helix to deform the membrane into elongated tubular peroxisomes. Such membrane deformation subsequently supports the accumulation of other fission factors including DLP1 and Mff at the Pex11pβ-enriched subregion (Itoyama et al., 2013), leading to fission of peroxisomes.

## Materials and Methods

### Lipids

1,2-dioleoyl-*sn*-glycero-3-phosphocholine (DOPC), 1,2-dioleoyl-*sn*-glycero-3-phosphoethanolamine (DOPE), L-α-phosphatidylinositol from bovine liver (PI), L-α-phosphatidylethanolamine-N-(lissamine-rhodamine B sulfonyl) (rhodamine-PE), 1-palmitoyl-2-docosahexaenoyl-*sn*-glycero-3-phosphocholine (PDPC), and 1-palmitoyl-2-docosahexaenoyl-*sn*-glycero-3-phosphoethanolamine (PDPE) were purchased from Avanti Polar Lipids (Alabaster, AL). Bovine brain L-α-phosphatidyl-L-serine (PS) was from Sigma (St Louis, MO). All lipids were stored in chloroform at −20°C.

### Antibodies

The antibodies used were rabbit antisera to Pex14p (Shimizu et al., 1999), PMP70 (Tsukamoto et al., 1990; Honsho et al., 1998), AOx (Tsukamoto et al., 1990), ADAPS (Honsho et al., 2008), and Pex5p (Otera et al., 2000). The antibody against Pex11pβ was raised against recombinant Pex11pβ protein (amino acid residues 1–203), then affinity-purified as described previously (Itoyama et al., 2012). Mouse monoclonal antibodies to human DLP1 (BD Biosciences, Franklin Lake, NJ), human tubulin (Abcam, Cambridge, UK), and c-Myc (Santa Cruz Biotechnology, Santa Cruz, CA) were purchased.

### Cell culture and siRNA transfection

HeLa cells were maintained in DMEM (Life Technologies, Grand Island, NY) supplemented with 10% FCS under 5% CO_2_. DNA transfection of HeLa cells was done with Lipofectamine (Life Technologies) according to the manufacturer's protocols. Knockdown experiments were performed using Stealth siRNA duplexes (Life Technologies). The siRNA target sequences were as follows: human Pex11pγ #1, 5′-AAGAGUCGCAAGAUGGUCCUGCAGU-3′; human Pex11pγ #2, 5′-UUGCUUAGUGUAGACAAACAUGGCC-3′; and human Mff, 5′-UUAUCACACUAGCAUUUGGAACUCC-3′. Stealth RNAi Negative Control (Life Technologies) was used as a control. The siRNAs for *PEX11α*, *PEX11β*, and *DLP1* were described previously (Itoyama et al., 2012). HeLa cells were trypsinized and plated on cover slips. Cells were immediately transfected with 20 nM of siRNA with Lipofectamine 2000 and cultured for 96 h.

### Plasmid construction

cDNA encoding human Pex11pβ was amplified by reverse-transcription PCR using mRNA from HeLa cells as the template. The PCR product was cloned into pcDNAZeo3.1 (Life Technologies) to generate the untagged Pex11pβ expression plasmid for HeLa cells. For the construction of His-tagged Pex11pα, the EcoRI-SalI fragment of Pex11pα was ligated into the corresponding sites of pCold1 (Takara, Japan). For His-Pex11pβ and His-Pex11pγ, the BamHI-EcoRI fragments were respectively amplified and inserted into pCold1. EGFP-fused Pex11pβ and Pex11pγ were generated by the insertion of the KpnI-BamHI fragment of plasmid encoding MBP and the BamHI-BglII fragment of plasmid encoding EGFP into the KpnI-BamHI site of the pCold1-Pex11pβ and pCold1-Pex11pγ, respectively. To construct EGFP-Pex11pα, using *MBP-EGFP* as a template, KpnI-BamHI fragment was amplified and ligated into pCold1 and the EcoRI-SalI fragment of the plasmid encoding Pex11pα was subsequently inserted into the plasmid. Introduction of point mutations in the N-terminal amphipathic helix of Pex11pβ was performed by site-directed mutagenesis using specific primers: Pex11pβ-P sense, 5′-TGAGCCTTGGAAGAAAGCTTCCACGC CTGGGTAACTCAGCACCTGCCCTTGAGTCAGCCAAAAG-3′; Pex11pβ-P antisense, 5′-CTTTT GGCTGACTCAAGGGCAGGTGCTGAGTTACCCAGGCGTGGAAGCTTTCTTC CAAGGCTCA-3′; Pex11pβ-φ sense, 5′-ACCTGAGCCTTGGAAGAAAGGAACTACGCGAGGGTAACTCAGAAGATGCCCTTG AGTCAGCCAA-3′; Pex11pβ-φ antisense, 5′-TTGGCTGACTCAAGGGCATCTTCTGAGTTAC CCTCGCGTAGTTCCTTTCTTCCAAGGCTCAGGT-3′. Pex11pβ-φ contains L58E, L61E, and A65E mutations, and Pex11pβ-P has point mutations to proline residues at L59 and D66. Pex14p was prepared as a GST-fusion as described previously (Itoh and Fujiki, 2006).

### Morphological analysis

For the observation of peroxisomal elongation by Pex11pβ, untagged Pex11pβ was expressed in HeLa cells. Cells were fixed at 6 h and 24 h after transfection. For the detection of peroxisomal fission induced by Pex11pα, Pex11pβ, Pex11pγ, and mutant forms of Pex11pβ, cells were transfected with the corresponding plasmids for 24 h. To verify the effect of these fission factors on Pex11pβ-dependent peroxisomal proliferation, Pex11p-expression plasmids were transfected into HeLa cells pre-treated for 48 h with siRNA for *DLP1* or *MFF*. Cells were fixed with 4% paraformaldehyde for 15 min at room temperature. Peroxisomes were visualized by indirect immunofluorescence staining with the indicated antibodies. Primary antibodies were detected with goat anti-mouse and anti-rabbit IgG conjugated to Alexa Fluor 488 or Alexa Fluor 568 (Molecular Probes/Life Technologies). Cells were observed under confocal laser microscopy (LSM710; Carl Zeiss, Oberkochen, Germany) with the Plan Apochromat 100 ×/1.3 NA objective lens. Line scanning was performed using the Plot profile tool in ImageJ software (National Institutes of Health, Bethesda, Md). For the analysis of number, size, and area of peroxisomes, images were taken of at least 30 cells by confocal microscopy. Each image was converted to a threshold image, and the number of peroxisomes was measured using the Analyze Particle command in ImageJ.

### *In silico* analysis

Multiple sequence alignment was prepared as described previously (Opaliński et al., 2011). The hydrophobic moments of the sequences of Pex11pα, Pex11pβ, and Pex11pγ were calculated using the EMBOSS server (http://mobyle.pasteur.fr/cgi-bin/portal.py?form = hmoment). The helical wheel representation of the Pex11p amphipathic region was prepared using the Heliquest server (http://heliquest.ipmc.cnrs.fr).

### Preparation of small unilamellar liposome

Liposomes were prepared essentially as described previously (Takei et al., 2001). The lipid mixture was dried under nitrogen stream to form a thin lipid film, and then rehydrated in Hepes-KOH buffer (20 mM Hepes-KOH, pH 7.4, 150 mM NaCl). Liposomes were formed by vortexing, then extruded by passing through a 100-nm filter 11 times. The lipid composition was designed to mimic mammalian peroxisomal membranes (Hardeman et al., 1990). DHA levels in human fibroblasts were determined (Itoyama et al., 2012). DHA liposomes contained DOPC/DOPE/PDPC/PDPE/PI/PS/Rhodamine-PE at a ratio of 29:25:25:11:5:5:1% (w/w).

### Protein purification

*E. coli* BL21(DE3) was transformed with pCold-Pex11p and protein expression was induced at 15°C for 24 h with 0.1 mM isopropyl β-D-thiogalactoside (IPTG). Cells were harvested and lysed in sarcosine buffer 50 mM Tris-HCl, pH 7.4, 0.5 M NaCl, 2 M Urea, 50 mM imidazole, 0.5% sodium N-lauroylsarcosine. After 15-min incubation on ice, the lysate was ultracentrifuged at 42,000 rpm for 30 min at 4°C using a Hitachi TLA100.3 rotor (Hitachi, Tokyo, Japan). The supernatant was incubated with Ni-NTA beads (Qiagen, Düsseldorf, Germany) at 4°C for 1 h, then the beads were collected and washed with wash buffer 50 mM Tris-HCl, pH 7.4, 150 mM NaCl, 0.5% sodium N-lauroylsarcosine. Protein was eluted by washing buffer containing 0.3 M imidazole. The elution buffer was used for protein reconstitution in the control of the experiment. For purification of MBP-EGFP-Pex11p proteins, cells were lysed in buffer containing *n*-dodecyl-β-D-maltoside (Dojindo, Kumamoto, Japan), and protein was affinity-purified by amylose resin (New England BioLabs, Hertfordshire, UK) according to the manufacturer's instructions. We named MBP-EGFP-Pex11p protein as “EGFP-Pex11p” and used as such, since the MBP-tag was not readily cleaved off by PreScission protease (GH Healthcare, Little Chalfont, UK).

### Protein reconstitution

Purified protein (up to 1 µg) or the same volume of the elution buffer was mixed with 50 µl of liposomes, and the mixture was frozen at −80°C for at least 5 min. Then, the mixture was thawed and diluted in 3 ml of Hepes-KOH buffer to form proteo-liposomes. Liposomes were collected by ultracentrifugation at 100,000 rpm for 1 h at 4°C, using a Hitachi TLA100.3 rotor. For the separation of protein precipitate after ultracentrifugation, the liposomal pellet was mixed with an equal volume of 60% sucrose (∼100 µl). One ml of 20% sucrose was added to the liposomes, and 100 µl of Hepes-KOH buffer was loaded on the top of the sucrose gradient. Then, the sample was centrifuged at 100,000 rpm for 1 h at 4°C using a Hitachi TLA100.3 rotor. Three serial fractions were harvested from the top of the tube by pipetting.

### Observation of protein-reconstituted proteo-liposomes

Precipitated proteo-liposomes were mounted on glass slides by mixing with PermaFluor (Thermo Scientific, Bremen, Germany), and covered with glass coverslips. Samples were observed under confocal laser-scanning microscopy with a Plan Apochromat 100×/1.3 NA objective lens. Photobleaching was performed in a 1-µm circle placed at the edge of a cluster of liposomes. Images were taken before and after 30 rounds of photobleaching, and the total fluorescence intensity of the liposomal clusters was analyzed. In the control experiment, an adjacent region to the liposome clusters was photobleached and subjected to analysis.

### Electron microscopy

For negative staining electron microscopy, proteo-liposomes were adsorbed onto a formvar-coated 300-mesh copper grid (Nisshin EM Co., Ltd, Tokyo, Japan) that had been glow-discharged just prior to use. The grid was negatively stained with 2% uranyl acetate and observed under a transmission electron microscope at 200 kV (Tecnai-20, FEI Company, Eindhoven, The Netherlands). To detect the localization of Pex11pβ, proteo-liposomes were stained with antibodies to Pex11pβ for 1 h at room temperature (RT), and labeled for 1 h with a goat anti-rabbit IgG secondary antibody conjugated to 10 nm gold particles (EY Laboratories, San Mateo, CA). The liposomes were then collected by ultracentrifugation and subjected to negative staining electron microscopy. For ultra-thin sectioning, liposomes were pelleted and fixed with 3% glutaraldehyde, and further processed for embedding in Epon and staining, as described previously (Meyerholz et al., 2005).

### Detection of mRNA by RT-PCR

Total RNA was extracted from HeLa cells using TRIzol reagent (Life Technologies), and cDNA was obtained by reverse transcription (RT), (PrimeScript RT-PCR kit; Takara, Tokyo, Japan) according to the manufacturer's instructions. RT-PCR was performed using a set of specific primers: Pex11pγ sense, 5′-TGATGACCTGGCCATGTTTGTCTA-3′, and antisense, 5′-AAGTGACAGCGCCTCCGACTGCAT-3′. Primer sets for Pex11pα and actin have been described previously (Itoyama et al., 2012).

## Supplementary Material

Supplementary Material
