# Supplementary material for: Pex11mediates peroxisomal proliferation by promoting deformation of the lipid membrane
Source: Biol Open. 2015 Apr 24;4(6):710–21. doi: 10.1242/bio.201410801 (PMC4467191; doi:10.1242/bio.201410801)
Supplement: Supplementary Material [file supp_4_6_710__index.html]

Pex11mediates peroxisomal proliferation by promoting deformation of the lipid membrane — Supplementary Material 

# Pex11mediates peroxisomal proliferation by promoting deformation of the lipid membrane

## bio.201410801 Supplementary Material

Yumi Yoshida et al. doi: 10.1242/bio.201410801

- Supplementary Material - Yumi Yoshida et al. doi: 10.1242/bio.201410801
