# Supplementary material for: Pex11mediates peroxisomal proliferation by promoting deformation of the lipid membrane
Source: Biol Open. 2015 Apr 24;4(6):710–21. doi: 10.1242/bio.201410801 (PMC4467191; doi:10.1242/bio.201410801)
Supplement: Supplementary Material [file bio.201410801_bio.201410801-s1.pdf]

Biology Open (2015) 1, 1–12 doi:10.1242/bio.201410801

Supplementary Material
Yumi Yoshida et al. doi: 10.1242/bio.201410801

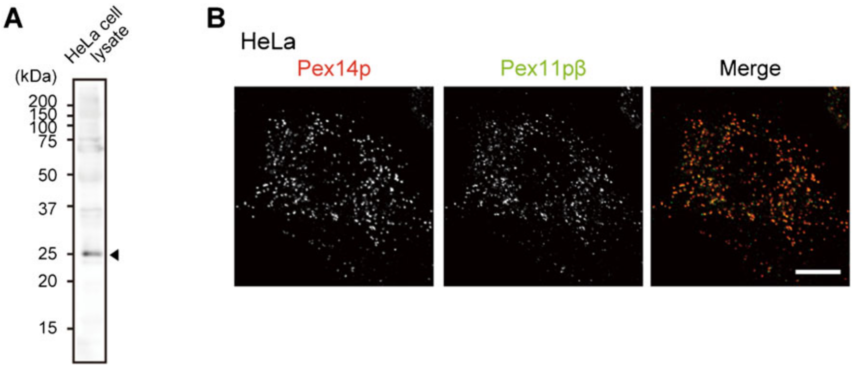

Fig. S1. Characterization of the antibody raised against Pex11pβ. (A) HeLa cells were lysed with SDS-PAGE sample buffer and subjected to western blotting. Pex11pβ (arrowhead) was detected using the anti-Pex11pβ antibody. (B) HeLa cells were fixed with paraformaldehyde and immunostained with the anti-Pex11pβ antibody in the presence of 0.1% saponin. Scale bar, 10 μm.

Biology Open

Biology Open **BIO_2015_10801.3d** 11/5/15 22:16:16
The Charlesworth Group, Wakefield +44(0)1924 369598 - Rev 9.1.480/W (Jul 30 2008)

Biology Open (2015) 1, 1–12 doi:10.1242/bio.201410801

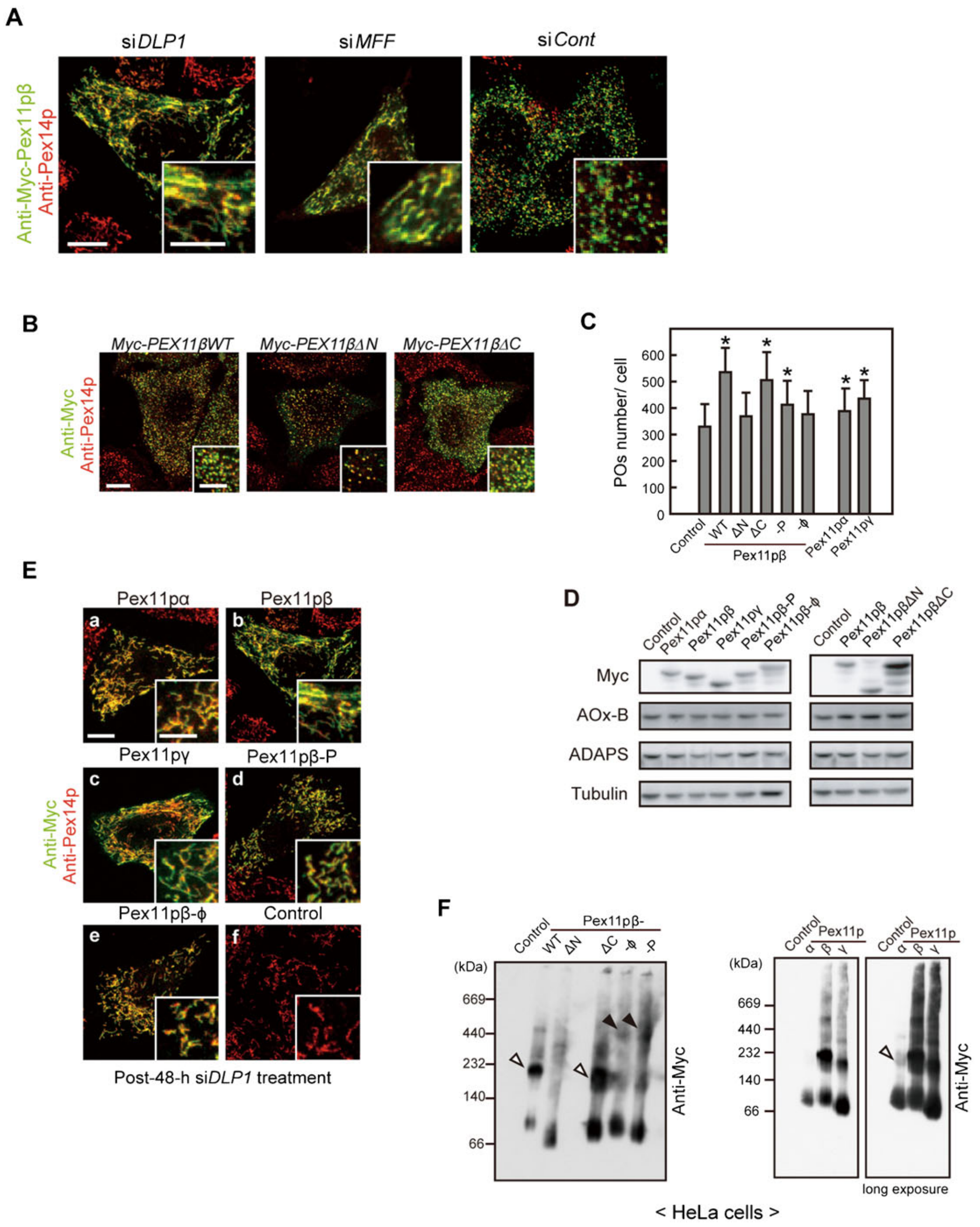

Fig. S2. Peroxisomal fission activity of Pex11p isoforms and mutated forms of Pex11pβ. (A) HeLa cells were treated with siRNA for *DLP1* or *MFF* for 48 h. The cells were then transfected with a cDNA encoding Myc-tagged Pex11pβ. Cells were fixed at 24 h after transfection, and immunostained with an anti-Myc antibody (green) and an anti-Pex14p antibody (red). Scale bars: 10 μm and 5 μm (inset). (B) Myc-tagged Pex11pβ truncation mutants were overexpressed in HeLa cells and immunostained with antibodies to Myc (green) and Pex14p (red). Peroxisomal fission was observed under confocal microscopy. Scale bars: 10 μm and 5 μm (inset). (C) Peroxisomal abundance per cell was determined in cells overexpressing Myc-tagged Pex11p in B and Fig. 3B. At least 20 randomly selected cells were analyzed. Data represent the means±SD. *$p<0.05$. (D) Cells were lysed and analyzed by SDS-PAGE and immunoblotting using the antibodies indicated on the left in the panel. (E) HeLa cells were treated with siRNA against *DLP1* for 48 h and transfected with the plasmids each coding for Myc-tagged proteins indicated in the panels (a-f). Cells were immunostained with antibodies to Myc (green) and Pex14p (red). Scale bars: 10 μm and 5 μm (inset). Note that while Pex11pβ and Pex11pγ resulted in extensive tubulation of peroxisomes, Pex11pα and the mutant forms of Pex11pβ resulted in a similar morphology to untransfected cells. (F) Pex11pα, Pex11pβ, Pex11pγ and the mutant forms of Pex11pβ were expressed in HeLa cells for 24 h. Cells were lysed with 1% digitonin and mixed with loading buffer (Life Technologies). The Pex11p oligomers were separated on a 4–16% native PAGE gradient gel (Life Technologies) and detected using an anti-Myc antibody. Open and solid arrowheads indicate oligomer formation in the active or inactive forms of Pex11p, respectively (left panel). For the detection of Myc-Pex11pα oligomer formation, a longer exposure of the film was required, as shown in the right panel.

Biology Open BIO_2015_10801.3d 11/5/15 22:16:17
The Charlesworth Group, Wakefield +44(0)1924 369598 - Rev 9.1.480/W (Jul 30 2008)

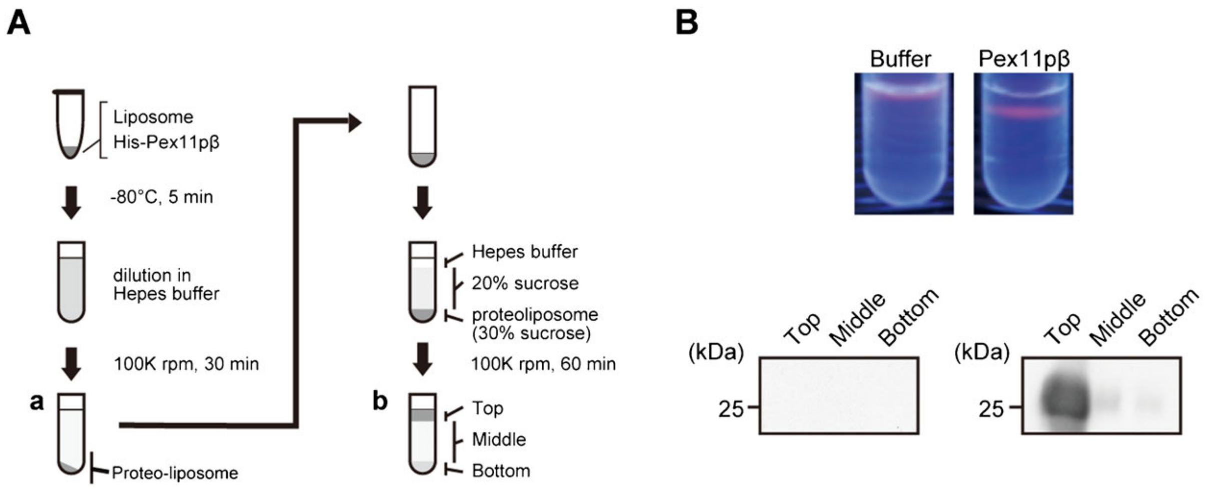

Fig. S3. Recovery of Pex11pβ proteo-liposomes. (A) Schematic representation of proteo-liposomes. Proteo-liposomes made after freezing and thawing were pelleted by centrifugation (a), then fractionated by ultracentrifugation over a sucrose gradient (b). (B) Recovery of liposomes after centrifugation. Liposomes were visualized by the incorporation of rhodamine-labeled PE. The majority of Pex11pβ was collected in the top fraction of the sucrose gradient.

Biology Open

Biology Open BIO_2015_10801.3d 11/5/15 22:16:25
The Charlesworth Group, Wakefield +44(0)1924 369598 - Rev 9.1.480/W (Jul 30 2008)

Biology Open (2015) 1, 1–12 doi:10.1242/bio.201410801

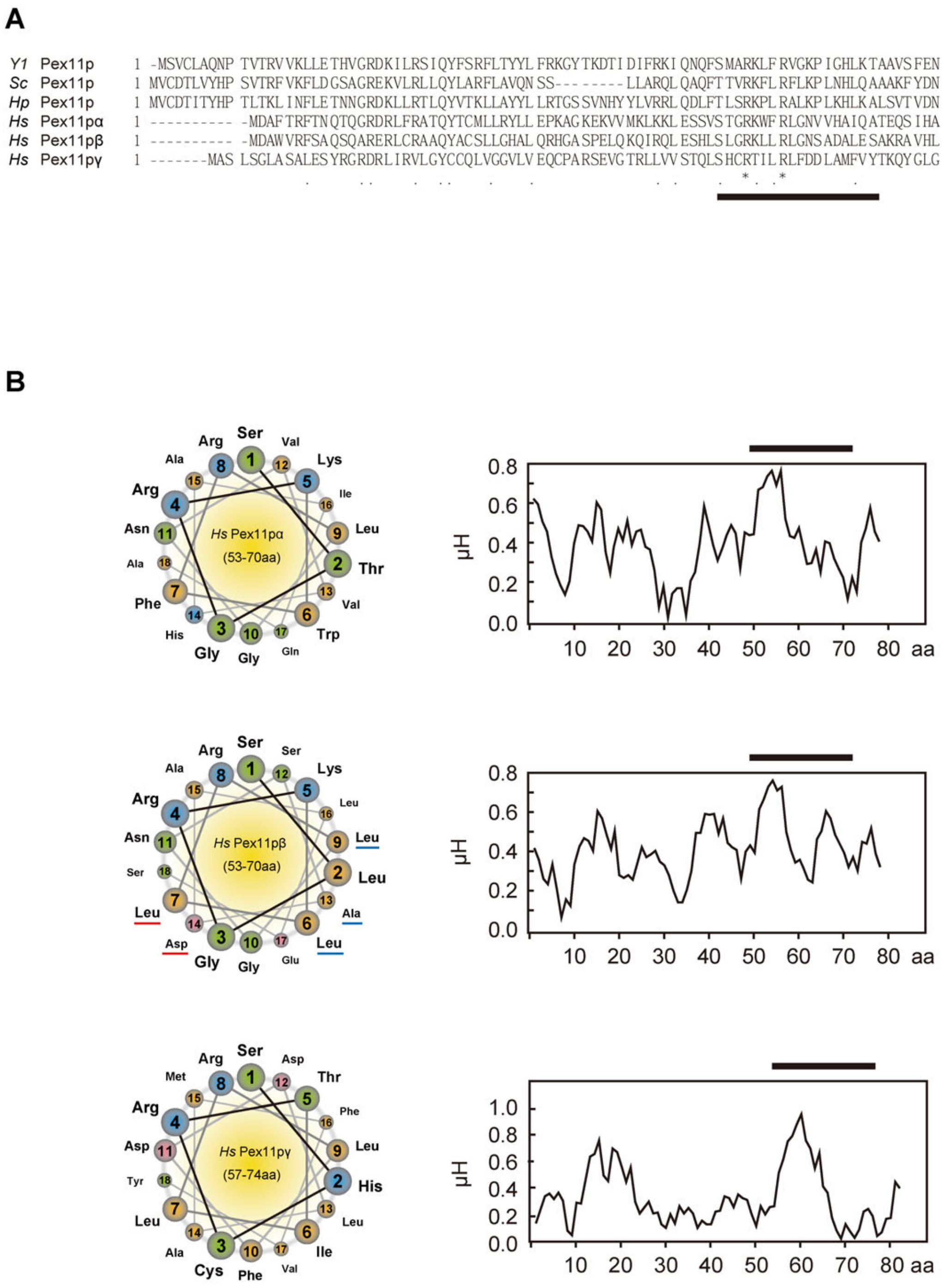

Fig. S4. Conserved amphipathic helix in human Pex11pα, Pex11pβ and Pex11pγ. (A) Multiple sequence alignment of N-terminal regions of Pex11p proteins from yeast and humans. Abbreviations and accession numbers: Yl-*Yarrowia lipolytica*, CAG81724; Sc-*Saccharomyces cerevisiae*, CAA99168; Hp-*Hansenula polymorpha*, DQ645582; hPex11pα-*Homo sapiens* Pex11pα, AAH09697; hPex11pß-*Homo sapiens* Pex11pβ, AAH11963; hPex11pγ-*Homo sapiens* Pex11pγ, AAH08780. The black line indicates that the conserved helix consists of hydrophobic and polar residues showing amphipathic properties. The asterisks in the sequence alignment indicate identical amino acids, and dots indicate amino acids with similar physicochemical properties. (B) Helical wheel representation of the portion of the amphipathic helix indicated in A. Amino acids are colored according to the physicochemical properties of the side chains (hydrophobic – yellow; polar, positively charged – blue; polar, negatively charged – pink; polar, uncharged – green). Hydrophobic moments (μH) for the N-terminal region were plotted. The conserved amphipathic helix is indicated by the black line, as in A. The lined region shows the highest hydrophobic moment in the N-terminal cytosolic region of all three isoforms.

Biology Open BIO_2015_10801.3d 11/5/15 22:16:27
The Charlesworth Group, Wakefield +44(0)1924 369598 - Rev 9.1.480/W (Jul 30 2008)

Biology Open
